# Levels of Toxic and Essential Elements and Associated Factors in the Hair of Japanese Young Children

**DOI:** 10.3390/ijerph20021186

**Published:** 2023-01-09

**Authors:** Emiko Kusanagi, Hitoshi Takamura, Nobuko Hoshi, Shing-Jen Chen, Mayumi Adachi

**Affiliations:** 1 Department of Childhood Education, Kokugakuin University Hokkaido Junior College, Takikawa 073-0014, Japan; 2 Department of Food Science and Nutrition, Faculty of Human Life and Environmental Sciences, Nara Women’s University, Nara 630-8506, Japan; 3 Department of Early Childhood Education, Junior College of Sapporo Otani University, Sapporo 065-8567, Japan; 4 Centers for Early Childhood Education and Care, Koen Gakuen Women’s Junior College, Sapporo 005-0012, Japan; 5 Research Group of Psychology, Graduate School of Humanities and Human Sciences, Hokkaido University, Sapporo 060-0810, Japan

**Keywords:** toxic element, essential element, hair, Japan, sex difference, yogurt, early childhood

## Abstract

There is growing concern regarding the effects of toxic element exposure on the development of children. However, little is known about the level of toxic elements exposure in Japanese children. The purpose of this study was to assess the concentrations of multiple elements (aluminum, cadmium, lead, calcium, copper, iron, magnesium, sodium, zinc) in the hair of 118 Japanese young children and to explore the factors associated with their element levels. The element concentration was analyzed by ICP-MS, and children’s food and water intake were assessed by the questionnaire. Results showed that there were no large differences between the level of elements in the hair of Japanese children and those of children in other developed countries. Girls had significantly higher levels of aluminum, copper, and iron (*p* = 0.000, 0.014, and 0.013, respectively), and boys had a higher level of sodium (*p* = 0.006). The levels of calcium, iron, magnesium, and sodium in nursery school children were significantly higher than those in kindergarten children (*p* = 0.024, 0.001, 0.046, and 0.029, respectively). Multiple regression analyses with controlling the confounding variables showed significant negative associations of frequency of yogurt intake with aluminum and lead levels (*p* = 0.015 and 0.037, respectively). When the children were divided into three groups based on the frequency of yoghurt consumption, viz. L (≤once a week), M (2 or 3 times a week), and H (≥4 to 6 times a week) group, the mean aluminum concentration (µg/g) in the L, M, and H groups was 11.06, 10.13, and 6.85, while the mean lead concentration (µg/g) was 1.76, 1.70, and 0.87, respectively. Our results suggested the validity of hair element concentrations as an exposure measure of essential elements and frequent yogurt intake as a viable measure for protecting children from toxic elements. However, these findings will need to be confirmed in more detailed studies with larger sample sizes in the future.

## 1. Introduction

There are global concerns about the adverse effects of toxic element pollution released by anthropogenic activities such as mining, factories, and e-waste processing on human health [1,2]. In particular, the adverse effects on the development of children need to be paid attention because of their vulnerability at a young age [3,4]. Regarding the exposure level to toxic elements in children, many countries have biomonitoring systems to assess the level of contamination in their bodies by measuring concentrations of essential as well as toxic elements in biological samples [5,6,7,8,9,10]. Despite the past experiences of element pollution in Japan, such as “Minamata disease” caused by methylmercury [11] and “Itai-itai disease” caused by cadmium [12], Japan does not have national biomonitoring systems [13], and there was a lack of data about the level of toxic elements exposure in Japanese adults, still less about children.

The purpose of this study is to assess young children’s exposure levels to essential elements (calcium (Ca), copper (Cu), iron (Fe), magnesium (Mg), sodium (Na), selenium (Se), and zinc (Zn)), as well as toxic elements (aluminum (Al), cadmium (Cd), and lead (Pb)). These toxic elements have been demonstrated to have adverse effects on children’s development. For example, exposure to Pb in childhood was associated with neurodevelopmental and behavioral problems [3,14,15]. Al was also suggested to have a possible relationship with developmental disorders such as autism [15,16]. Hg and Cd have been shown to have an adverse effect on children’s neuropsychological development [17,18,19].

In biomonitoring assessing the levels of elements exposure, biological samples such as blood, urine, and hair have generally been used [6,9,20,21]. Whereas levels of elements in blood and urine are temporary exposure measures, those in hair are retrospective and reflect long-term exposure depending on hair length, as hair is not affected by metabolic pathways inside the body after growing outside the skin [22,23]. Moreover, hair is sampled noninvasively and is easily stored. There have been an increasing number of studies measuring multiple elements in hair in non-contaminated as well as contaminated areas in recent years [5,24,25,26,27,28,29]. In this study, we used children’s hair to assess the exposure levels to elements.

The second purpose is to explore possible factors that might affect the exposure level of these elements in children’s hair. The design of this study was directed by the following considerations.

First, we expected the sex and age of children to associate with the level of elements in their hair [5,20,30,31,32]. Secondly, we expected the type of institution (nursery school or kindergarten) might also be an important factor, as the availability of nutritionally controlled lunch varies by institution the child attends. Japanese young children usually go to either nursery school or kindergarten. Nursery school children are provided with nutritionally controlled lunches and snacks, whereas kindergarten children eat nutritionally uncontrolled lunches prepared by parents. Based on these facts, we expected there might be a possibility that essential elements (i.e., mineral) levels in nursery school children are higher than those of kindergarten children. Thirdly, as food and water intake are naturally expected to be associated with exposure level to elements [33,34,35,36], we considered it necessary to include the intake of these items. In addition, there is the finding that foods are not only a source of exposure to elements, but that specific foods reduce the amount of exposure to toxic elements. For example, the Lactic acid bacteria contained in yogurt has showed to suppress the toxic element levels in animal and in vitro experiments [37,38,39]. We considered it important for us to confirm the conductive effect of yogurt intake for suppressing toxic element levels in Japanese children. Children’s food and water intake was assessed by the questionnaire survey. Moreover, in the questionnaire, we asked mothers about the environment around their house, which is supposed to be an important source of element exposure.

In summary, the purpose of this study was to measure the concentrations of multiple elements in the hair of Japanese young children and to explore the factors associated with their element levels.

## 2. Materials and Methods

### 2.1. Study Population

In 2012, the research was conducted through kindergartens and nursery schools with which we, and our acquaintances, had some connections. These kindergartens and nursery schools are located in 5 regions of Japan (the prefectures of Yamaguchi, Nara, Yamanashi in the mail islands, *Honshu*, and the cities of Sapporo and Takikawa in the northern island, *Hokkaido*). Parents of children aged 3–6 years from kindergartens and nursery schools were asked to answer a questionnaire concerning their children’s demographic information, food intake, types of drinking and cooking water, and the environment surrounding their houses. While 1351 questionnaires were distributed, 872 answers were obtained (return rate = 64.5%). Among the 202 mothers who initially consented to contribute a sample of their children’s hair without any chemical treatment (stains and/or permanent waves), we only managed to obtain 118 samples due to various reasons (e.g., parents changing their minds, incomplete questionnaire). The characteristics of children and their parents are shown in Table 1.

Prior to parents’ answering the questionnaire and to hair sample collection from the children, the objectives and methods of this study were explained and informed consents in written form were obtained from them.

### 2.2. Questionnaire

Parents were asked to complete a questionnaire concerning their demographics, food consumption, types of drinking and cooking water, and the environment surrounding their houses. In relation to food consumption, the following items from The Food Frequency Questionnaire (used in Hokkaido Birth Cohort Study [40] were included: milk, yogurt, cheese, butter, eggs, fatty fish (sardine, mackerel, salmon, saury, yellowtail, herring, toro of tuna, etc.), non-fatty fish (trout, white-fleshed fish, bonito, red part of tuna, etc.), and other seafood (squid, octopus, shrimp, crab, shellfish (clam, freshwater clam, etc.), green and yellow vegetables (spinach, carrot, tomato, bell pepper, pumpkin, broccoli, etc.), and other vegetables (onions, cabbage, radish, Chinese cabbage, cauliflower, etc.). For all items, the frequency of consumption during the past year was questioned, with 8 response options provided (i.e., almost never, once a month, 2–3 times a month, once a week, 2–3 times a week, 4–6 times a week, once a day, and at least twice a day).

Concerning the drinking and cooking water, mothers were allowed to choose from one or two of the following water categories for drinking and cooking, respectively: tap water, filtered tap water, well water, commercially available Japanese water, commercially available foreign water, and deep sea water. For each item of water, a score of 1 was assigned, and when no selection was made, a score of 0 was assigned.

With respect to the environment surrounding their houses, mothers were asked to choose one or more from the following items: residential area, shopping district, rural area, fishing village area, factory area, downtown area, heavy traffic, heavy construction, and near railroad track.

### 2.3. Assessment of the Hair Concentration of Elements

Because we wanted hair samples that reflected the children’s food consumption during the previous year, we instructed the mothers to collect their children’s hair (1 g) between the root and 12 cm from the scalp, based on the assumption that children’s hair grows at the rate of 1 cm per month. Mothers were provided with detailed instructions for harvesting their children’s hair and for returning the sample by post. This procedure has been described in detail elsewhere [41]. The returned samples were stored at room temperature. The samples were prepared in a fume hood in order to avoid external contamination. The collected hair samples were washed with acetone and distilled water and then dried naturally. A part of the treated hair was accurately weighted with an accuracy of 0.1 mg (approximately 200 mg), dissolved in 4 mL of 70% HNO_3_ acid (Nitric Acid (1.42) for Ultratrace Analysis, Wako Pure Chemical Industries), and ashed at 130 °C for 20 h, and the volume was fixed at 10 mL by adding HNO_3_ acid and ultrapure water. The element concentration in each sample was analyzed on an Agilent (Santa Clara, CA, USA) ICP-MS7500i using Co, In, and Tl as the internal standard elements. The human hair reference material, NIES CRM No. 13 (National Institute of Environmental Studies, Tsukuba, Japan), was used for the laboratory quality control and the validation of results. The recovery rate of each element of standard hair is as follows: Al (58.7%), Ca (94.5%), Cd (121.7%), Cu (94.8%), Fe (72.6%), Mg (80.9%), Na (79.5%), Pb (107.0%), Se (174.3%), and Zn (102.7%). The recovery rate of Al was lower than that of the other elements, possibly due to passivation. Because the recovery rate of Se was much above 100%, Se was excluded from the following analyses. Analytical precision was estimated from triplicate analyses of every sample and the mean value was used. The Relative Standard Deviation (RSD) for all elements except Al, Cd, and Hg were less than 10%. The number of samples with an RSD greater than 10% was two (13.9% and 25.1%) for Al, one (14.1%) for Hg, and nine (11.9 to 96.0%) for Cd.

### 2.4. Statistical Analysis

Because of the skewed distributions of the concentration of each element in the hair, the concentration of each element in the hair was log-transformed for statistical analysis. Given that the log-transformed values of Na and Fe concentrations in the hair of one child were outliers, that is, higher than the Q3 (the third quartile) + 3 × (Q3 − Q1), these values were excluded from the analysis. There was no outlier in other elements. Prior to statistical analysis, distribution of each log-transformed element was assessed to be normal from each histogram. As concentrations of Cd were below the limit of detection (0.0294 µg/L) in a high proportion of the sample (85.6%), they were categorized as detected (1) or undetected (0) in the correlation analysis. All food consumption frequencies were converted to times per week, and then were log-transformed, in which the category “almost never” was assumed to have a value equal to half of “once a month” (0.125/week). Dichotomous variables such as sex, types of children’s daycare facilities, and usage of tap water with or without filter were transformed into dummy variables (girls = 0, boys = 1; nursery school = 0, kindergarten = 1; without filter = 0, with filter = 1). Principal component analysis was performed to explore the interrelationships among levels of element in hair and component scores were calculated. Student’s *t*-test was used to determine the existence of differences in each component score and element exposure level.

To explore the decreasing effect of yogurt intake on toxic element exposure levels, multiple regression analysis and analysis of variance were conducted on the toxic element concentrations in hair. All analyses were carried out using SPSS (version 26). The statistical significance level was set at 0.05 (*p* < 0.05).

## 3. Results

### 3.1. Element Concentrations in the Hair of Japanese Children

Summary statistics describing element concentrations in children’s hair are shown in Table 2. The order of the concentration of elements in hair was Ca > Na > Zn > Mg > Cu > Al > Fe > Pb > Hg > Cd.

### 3.2. Interrelationships among Elements Concentrations in Children’s Hair

**Figure 1:**
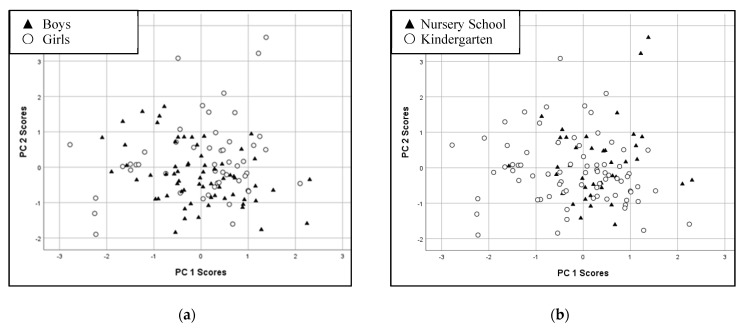
Score plots from the principal component analysis of 117 children’s sample: (**a**) Boys and Girls; (**b**) Nursery school and Kindergarten. One child with outliers for Na and Fe concentrations was excluded from the principal component analysis.

To investigate the interrelationship of the hair element levels, we performed principal component analysis (PCA) on the correlation matrix of log-transformed elements data. Prior to the execution of PCA, appropriateness of data was ascertained through KMO and Bartlett test of Sphericity. Although there were three components with eigenvalues exceeding 1, an inspection of the scree plot revealed a clear break after the second component. The two-component solution was further supported by the results of parallel analysis, which showed only two components with eigenvalues exceeding the corresponding criterion values for a randomly generated data matrix of the same size. The two-component solution explained a total of 49.2% of the variance, with Component 1 contributing 29.3% and Component 2 contributing 19.9%. To aid in the interpretation of these two components, varimax rotation was performed. The main loadings on component 1 were Mg, Ca, and Zn, that is, the essential major elements (Table 3). The main loadings on component 2 were Pb, Al, Fe, Cu, and Cd. Hg and Na had loadings lower than 0.4 on both components 1 and 2. Based on this PCA, we calculated principal component scores for each child. These scores are shown by boys and girls (Figure 1a) and by nursery school and kindergarten children (Figure 1b).

Student’s *t*-test was conducted to compare the component 1 and 2 scores between boys and girls and between nursery school and kindergarten children. There was significant difference in component 1 scores between nursery school children (mean = 0.28, SD = 0.78) and kindergarten children (mean = −0.18, SD = 1.05; *p* = 0.008, two-tailed). Nursery school children had higher scores in component 1, on which, essential elements had high loading values. For component 2, girls (mean = 0.25, SD = 1.13) had higher scores than boys (mean = −0.21, SD = 0.82; *p* = 0.012, two-tailed).

### 3.3. Associations of Children’s Hair Element Level with Demographics, Food and Water Intake, and Environment around Their House

Among the demographic, food intake, and drinking and cooking water variables, only variables which significantly related to any log-transformed value of element concentration were shown (Table 4). Among the variables concerning drinking and cooking water, those chosen by less than 10% of the respondents were excluded from further analysis. Concerning the environment around children’s houses, most of their houses were located in residential areas (72%) and the other options were selected no more than 10% of the time, except heavy traffic (35.9%). With respect to “heavy traffic,” since subjective factors seemed to have influenced some mothers’ judgement of the heaviness of traffic in the surroundings of their houses, we decided to exclude all these environment variables from analysis. The sex of the children and the type of facilities that children attend had significant correlations with some element levels, as expected from the result in the principal component scores. Girls had significantly higher levels of Al, Cu, and Fe, and boys had higher levels of Na. The hair levels of Ca, Fe, Mg, and Na in nursery school children were higher than those in kindergarten children. These results were confirmed by the *t*-test for each log-transformed element concentration of hair between boys and girls and between nursery school and kindergarten children (Table 5).

Children’s age had a significant relation with the log-transformed Pb hair concentration; younger children had higher levels of Pb than older children. There were significant relations between paternal education level and Na, and between family income and Cd and Cu, whereas body weight, parental ages, and maternal education were not significantly associated with any element concentration in children’s hair.

The log-transformed yogurt intake frequency showed significant negative relations with the log-transformed concentrations of Al, Cu, and Pb, but not with those of Cd and Hg. The log-transformed concentration of Al was also significantly associated with cooking tap water and cooking filtered tap water, but not with any kinds of drinking water. The log-transformed concentration of Cu was also related to the log-transformed intake frequency of cow milk. The log-transformed concentration of Ca and Mg showed significant positive associations with consumption of “green and yellow vegetables”, which contain a lot of these minerals. The log-transformed concentration of Ca was also related to the log-transformed non-fatty fish intake frequency.

To further clarify the effect of yogurt consumption on reducing the exposure levels of toxic elements, that is, Al and Pb, multiple regression analysis was performed. Due to the relatively large number of controlling variables as compared with the number of samples, only the demographic variables significantly associated to any of the two toxic elements in the correlation analysis. Consequently, only sex and age were entered as the controlling variables. In addition, in the regression model of Al, only tap water was entered into the model to avoid muticollinearity, because correlation between tap water and filtered tap water in the cooking were high (*r =* −0.73). The log-transformed yogurt intake frequency still had a significant negative effect on log-transformed concentrations of Al and Pb in children’s hair after controlling these variables (Table 6).

In order to explore the reducing effect of yogurt intake on exposure level of Hg, regression analysis was performed entering sex, age, and foods with significant correlations with log-transformed concentration of Hg as the controlling variables. Yogurt intake had no reducing effect on exposure level of Hg and only fatty fish were significantly associated with log-transformed Hg level. As most of the concentrations of Cd were below the limit of detection, regression analyses on Cd were not executed.

**Figure 2:**
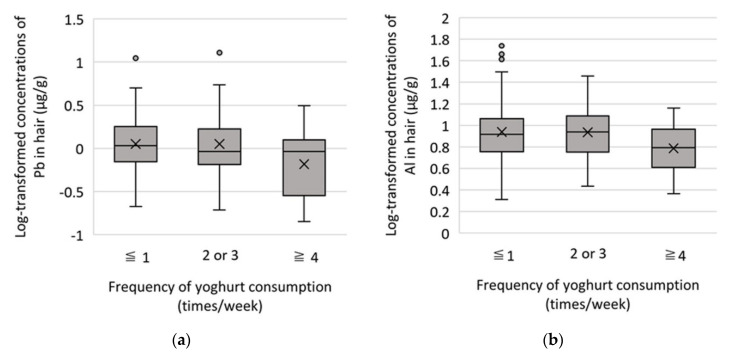
Boxplots showing log-transformed elements concentration of children’s hair in each frequency of yogurt consumption (times/week): (**a**) Pb; (**b**) Al. A black bar indicates the median and a black × mark the mean.

To ascertain the minimal frequency of yogurt consumption required to obtain a significant reduction of concentration levels of Al and Pb, subjects were divided into three groups: L group (≤once a week), M group (2 or 3 times a week), and H group (≥4 to 6 times a week). Analysis of variance was performed to examine the differences between log-transformed Al and Pb concentrations across these groups. Significant effects from yogurt consumption were observed for log-transformed Al [*F* (2, 114) = 3.65, *p* = 0.029] and Pb concentrations [*F* (2, 114) = 4.16, *p* = 0.018]. The results of the post-hoc comparison indicated that the mean of the log-transformed Al concentrations in the H group was significantly lower than that in the L group, and the mean of the log-transformed Pb concentrations in the H group was lower than that in the M and L groups (Figure 2). The mean Al concentrations in the L, M, and H groups were 11.06 µg/g, 10.13 µg/g, and 6.85 µg/g, while that of Pb was 1.76 µg/g, 1.70 µg/g, and 0.87 µg/g, respectively.

## 4. Discussion

To the best of our knowledge, this is the first study since studies performed over 30 years ago [42,43] to assess the levels of multiple elements including toxic elements in Japanese children’s hair. The element concentrations of past Japanese and those from children of other countries are shown in Table 7. Most of the element concentrations in Japanese hair, except for Al and Cu, have decreased over the last few decades, as compared to past Japanese data, including that of adults [42,43]. Compared to the level of elements in children’s hair in other developed countries, levels of Zn, Fe, and Cd in the hair of Japanese children were lower than those of most of the other developed countries [5,24,44,45,46], and levels of Cu, Hg, Pb, and Zn were similar to those of Spain [47], where data on some elements were not available. Thus, there was no large difference between the level of elements in the hair of Japanese children and those of children in other developed countries. This might be due to the globalization of eating habits.

An additional point worth mentioning is that there is a certain pattern in sex difference in hair element levels. Our results showed that Al, Cu, and Fe levels in girls were higher than those in boys, whereas the Na levels in boys’ hair were higher than those in girls’ hair. Table 7 showed the sex difference in levels of elements of children’s hair in other countries. All significant sex differences in Cu and Fe in other countries showed that girls’ levels were higher than boys’ [5,20,24,27,31,47]. Sex differences in Al levels in other countries were also similar to our result, except for Germany, in which boys had higher Al levels than girls [20,45,47]. Although there were limited studies measuring Na concentration in children’s hair, boys had higher Na levels than girls in all studies reporting significant sex differences in Na, including contaminated areas [5,31]. Whereas no sex differences in Ca, Mg, and Zn levels in hair were observed in our results, Ca and Mg levels in girls were higher than those in boys in all studies of other countries (Germany, Italy, Russia, and Bangladesh) reporting significant sex differences in Ca and Mg [20,31,44,46,52], and Zn levels in girls were also higher than that in boys, except for a study in Korea [5,27,45,51]. In other biological samples in previous studies, sex differences in essential elements’ concentrations have been shown to be higher in girls in general [56]. On the other hand, concerning the studies showing significant sex differences in Pb and Cd, boys had higher Pb levels than girls [5,20,29,51] and girls had higher levels of Cd than boys, except one study, respectively [20,24,27,31].

There are several possible causes of sex differences. One is the difference in behavioral access to the source of exposure. For example, boys may have more opportunities to come into contact with toxic elements in soil because they are more active and play outdoors more often than girls [45,57]. Other explanations are due to the sex differences in eating habits and diets [21] and/or growth rates [58]. In addition, still unknown hormonal systems are suggested to influence sex differences in element levels in hair [59]. The results that show girls are more exposed to essential elements and boys are more exposed to toxic elements might be related to the biological vulnerability of boys [60]. Children’s age had significant relation only with Pb level: younger children had higher levels of Pb than older children, which is consistent with previous reports in other countries [32,50,59]. Exposure sources of Pb include factory, e-waste processing, some home paints, drinking water through older lead pipes, and leaded gasoline emission [61]. The former two are unlikely in the area where our sample was gathered. Paints had not been used in most of the Japanese houses. Thus, leaded gasoline emission and old lead water pipes are more likely to be the sources of Pb exposure in Japanese children.

The Pb in ambient air and the exposure amount of humans to Pb was globally reduced by more than half, due to the wider use of lead-free gasoline by the end of 2000 [62,63,64]. As Japan had completed the non-lead gasoline system in 1983 [65], the Pb levels in hair in our sample were reduced to less than half that of adults in the 1980’s (Table 7). However, Pb emitted from vehicles before the completion of non-lead gasoline systems had contaminated and remained on topsoil. Takaoka et al. [66] measured Pb concentration in the surface soil on playgrounds in Tokyo and concluded that gasoline Pb from the past contributed to increased Pb concentration in the surface soil.

As young children are close to the ground and easily inhale fugitive dust containing Pb, and often bring their unwashed hands to their mouths, younger children might have higher Pb exposure than older children, a conclusion consistent with our results and similar results obtained in other countries [24,50,59]. When a child unintentionally ingests 200 mg of soil a day, the amount of that child’s intake of Pb from the soil was estimated to exceed their Pb intake from food [67]. Mielke et al. [68] also indicated that Pb in topsoil is an important factor in children’s Pb exposure, based on the survey in metropolitan New Orleans.

In addition, drinking water supplied through aged lead pipes in old houses could be another source of exposure. Whereas most of the water pipes that distribute water from water treatment facilities have been replaced by pipes made from materials other than lead by local governments, the water pipes that lead from the distributing pipes to the faucets in the house are privately owned. These aged lead pipes remain in use in old houses, which might be a source of higher levels of Pb in children’s hair.

As expected from the difference in nutritional management of meals eaten in nursery schools and kindergartens, nursery school children’s hair had higher levels of elements, especially essential elements. A nursery school is a facility for children of working mothers, and is under the supervision of the Ministry of Health, Labor and Welfare. It provides children with lunch and snacks, following prescription. For example, it is stipulated that nursery schools should provide one third of the required daily mineral intake for children in lunch and about 10–20% in snack. On the other hand, kindergarten is under the supervision of the Ministry of Education, Culture, Sports, Science and Technology, and there is no national stipulation concerning the dietary contents of kindergarten lunches. Thus, children in some kindergartens eat lunch boxes made by their parents, and in other kindergartens, children eat outsourced lunches. Accordingly, nursery school children could be in a more ideal nutritional condition than kindergarten children, a fact that could explain the results obtained in this study, i.e., nursery school children having higher levels of these minerals.

In addition, the result showing that loadings of Hg on both components 1 and 2 were lower than 0.4 in PCA implies that potential source of Hg could be different from exposure sources of elements loading component 1 and 2. This coincides with the results showing that the main exposure source of Hg was reported to be fatty fish in Kusanagi et al. [41]. Although some researchers have doubts about the validity of hair concentration as an exposure measure [52,69,70], our results suggest that hair element analysis is an effective tool for nutritional assessment, even in developed countries, as has been demonstrated in the study of nutritional intervention by WEP and the government program in Tajikistan [71].

Finally, the result showing that yogurt intake frequency was significantly negatively related to the concentrations of Al and Pb seems to be important. This suggested that yogurt intake might have a beneficial effect of suppressing the absorption of Pb and Al into children’s bodies and reducing exposure levels of these toxic elements in children, even in a developed country like Japan. In addition, it was suggested that consumption of yogurt four or more times a week could reduce the exposure level to Pb by about half and Al exposure level by about 40% when compared to a group consuming yogurt less frequently. Although removing the sources of toxic elements would logically be the priority measure to take, such a measure can be both costly and time consuming. Thus, before this can be achieved, proposing frequent intake of yogurt should be considered as a viable alternative for reducing the harmful effects of toxic elements, as our results would suggest.

In recent years, there have been extensive studies on fermented foods like yogurt as probiotics, as evidenced by a recently established view that the intestinal microbiota is an interface between food and health [38,72,73]. Lactobacillus contained in yogurt has been demonstrated to inhibit the uptake of toxic elements like Pb, Cd, Hg, and Al, and promotes their excretion by many kinds of mechanisms: binding elements to their cell surfaces [74,75,76,77], converting toxic elements to a less toxic form [38], and preventing the disruption of the human intestinal barrier [78].

While these studies examining the mechanism are confined to experiments on animals or in vitro, few studies have examined the effect of yogurt in the actual human body. To our best knowledge, only the study by Bisanz et al. [79] that addressed the effects of yogurt in a human population, with a particular focus on pregnant women and children, showed the effects of consumption of Lactobacillus in reducing blood levels of Hg and As in pregnant women, but no effect on Pb level in pregnant women nor on toxic elements in children. While the result of the present study showed that yogurt consumption has an effect of reducing hair levels of Pb and Al, there was no association between yogurt consumption and Hg and Cd levels in our results. Hg levels in the blood of pregnant women and children in the study by Bisanz et al. noted above were below half of those of Japanese [80]. Our finding of a non-significant association between yogurt consumption frequency and Hg exposure level might suggest that the conductive effect of yogurt intake depends on the level of Hg exposure.

Another important finding of the present study was that after controlling other demographic variables, children in homes that used tap water for cooking showed higher Al concentration levels than children whose homes did not use tap water for cooking. Al is contained in tap water, as aluminum salts are used worldwide as coagulants in water treatment facilities. Our survey also showed that 80% of households that did not use tap water for cooking used filtered tap water through a water purifier, which may account for the lower Al level found among children coming from households that did not use tap water. From the calculation of the *β* value (0.22) of tap water usage in the regression analysis, the contribution of tap water consumption to Al exposure level was estimated to be 4.8% of the total intake variance. This estimation was consistent with the description of the World Health Organization [81] that the contribution of drinking water to the total oral exposure to Al is usually less than 5% of the total intake.

Needless to say, this study has limitations. First, we investigated only four toxic and six essential elements in children’s hair; most concentrations of Cd in hair were below the limit of detection, and the recovery rates of Al and Se were worse. In order to provide reference values of elements for Japanese children’s hair and to obtain clearer conclusions about factors affecting element levels, assessments of more elements with a larger sample size under conditions that allow measurement to lower concentrations of trace elements are needed. Second, to examine the associated factors with the concentrations of elements in hair, we only asked about the approximate frequency of food and water intake in the questionnaire, which could have contributed to the low correlation coefficients between intake of food and water and elements level in hair. In addition, mother’s answers might be affected by their memory biases. It would be necessary to examine dietary intake in detail and/or assess the concentrations of elements in multiple meals eaten by children and in tap water and filtered water at home. Finally, because the degree of effect of yogurt in suppressing the absorption of toxic elements might depend on the type of lactic acid bacteria, it will be necessary to specify the type of lactic acid bacteria in the yogurt consumed by children, and to examine the association between the type of lactic acid bacteria and the effects of reducing the levels of toxic elements in future studies.

## 5. Conclusions

There were no large differences between the levesl of essential (Ca, Fe, and Mg) and toxic elements (Al and Pb) in the hair of Japanese children and those of children in other developed countries. Sex is an important factor affecting hair element concentrations, suggesting that there is a certain pattern with respect to sex differences. The higher concentrations of essential elements in nursery school children who were provided with a nutritionally controlled diet than in kindergarten children who were not, suggested the validity of hair element concentrations as an exposure measure, at least for these essential elements. It is also suggested that frequent intake of yogurt may reduce exposure levels to Pb and Al in children in a developed country like Japan; therefore, suggesting frequent intake of yogurt could be an important strategy for protecting children from these toxic elements. However, more detailed studies with larger sample sizes are required in the future to confirm our results.

## Figures and Tables

**Table 1:** General characteristics of children and their parents.

	*n*	%
Total	118	
Region		
Yamaguchi	16	13.6
Nara	18	15.3
Yamanashi	30	25.4
Sapporo	34	28.8
Takikawa	20	16.9
Sex		
Boys	68	57.6
Girls	50	42.4
Children’s age		
3 year olds	27	22.9
4 year olds	39	33.1
5 year olds	43	36.4
6 year olds	9	7.6
Type of children care/education institution		
Nursery School	42	35.6
Kindergarten	76	64.4
Father’s age (year)	38.6 ± 5.4	
Father’s education		
<High school	1	0.8
High school	37	31.4
Technical/Junior college	25	21.2
University	46	39.0
>University	7	5.9
Missing	2	1.7
Mother’s age (year)	36.6 ± 4.7	
Mother’s education		
<High school	2	1.7
High school	37	31.4
Technical/Junior college	54	45.8
University	24	20.3
>University	1	0.8
Annual family income (yen)		
<2,000,000	5	4.2
2,000,000~3,000,000	3	2.5
3,000,000~5,000,000	49	41.5
5,000,000~7,000,000	36	30.5
7,000,000~10,000,000	17	14.4
>10,000,000	2	1.7
Missing	6	5.1
Drinking water		
Tap water	68	57.6
Filtered tap water	37	31.4
Well water	1	0.8
Commercially available Japanese water	21	17.8
Commercially available foreign water	1	0.8
Deep sea water	0	0.0
Missing	1	0.8
Cooking water		
Tap water	82	69.5
Filtered tap water	34	28.8
Well water	1	0.8
Commercially available Japanese water	6	5.1
Commercially available foreign water	1	0.8
Deep sea water	0	0.0
Missing	1	0.8

**Table 2:** Concentration of elements in the hair of Japanese children.

Element	*n*	GM ^a^	Minimum	25 Percentiles	Median	75 Percentiles	Maximum
Al (μg/g)	118	7.98	2.04	5.41	8.17	10.78	54.86
Ca (μg/g)	118	270.57	46.06	176.04	279.52	407.13	1514.63
Cd (μg/g) ^b^	118	0.001	0.0004	0.0007	0.0009	0.0011	0.797
Cu (μg/g)	118	11.01	3.95	7.75	9.95	13.94	44.15
Fe (μg/g) ^d^	117	7.24	2.61	5.47	7.09	8.91	30.56
Hg (μg/g) ^c^	118	0.87	0.17	0.54	0.88	1.26	3.20
Mg (μg/g)	118	26.75	4.34	16.56	25.83	40.70	150.57
Na (μg/g) ^d^	117	89.67	6.35	48.54	88.20	182.62	4590.57
Pb (μg/g)	118	0.98	0.14	0.62	0.96	1.49	12.85
Zn (μg/g)	118	49.62	10.91	39.60	51.07	64.22	185.17

^a^ GM: Geometric mean. ^b^ Values below the detection limit were set at half of the limit of detection and treated as real values. ^c^ Data comes from Kusanagi et al. [41]. ^d^ Outlier was excluded.

**Table 3:** Pattern for principal component analysis with varimax rotation of two factor solution of log-transformed concentrations of children’s hair elements.

Element	Pattern Coefficients		Communalities
	Component 1	Component 2	
Mg	**0.908**	0.081	0.832
Ca	**0.905**	0.167	0.847
Zn	**0.586**	0.039	0.344
Na	0.385	−0.134	0.166
Hg	0.315	0.083	0.106
Pb	−0.088	**0.774**	0.607
Al	0.104	**0.771**	0.606
Fe	0.271	**0.663**	0.513
Cu	0.338	**0.605**	0.480
Cd	−0.254	**0.592**	0.415

Extraction method: Principal component analysis. Rotation method: Varimax with Kaiser Normalization. Loadings with higher than 0.40 are bolded.

**Table 4:** Pearson correlations between log-transformed hair elements’ concentrations and demographic and food and water consumption (*p* values in parentheses).

	Al	Ca	Cd	Cu	Fe	Hg	Mg	Na	Pb	Zn
Demographics										
Sex ^a^	−0.33	−0.04	0.11	−0.23	−0.25	0.04	−0.03	0.25	0.00	−0.04
(*n* = 118)	(0.000)	(0.662)	(0.246)	(0.014)	(0.007)	(0.679)	(0.737)	(0.006)	(0.987)	(0.643)
Age	0.03	0.01	−0.16	0.10	−0.04	0.02	0.02	0.00	−0.20	0.16
(*n* = 118)	(0.764)	(0.905)	(0.088)	(0.288)	(0.705)	(0.851)	(0.876)	(0.984)	(0.034)	(0.093)
Paternal education	−0.06	0.09	−0.10	0.08	−0.09	0.19	0.10	0.26	0.06	−0.05
(*n* = 116)	(0.531)	(0.322)	(0.302)	(0.376)	(0.354)	(0.047)	(0.301)	(0.005)	(0.533)	(0.590)
Family income	−0.07	0.02	−0.22	0.24	−0.09	0.28	−0.01	0.03	−0.06	−0.02
(*n* = 112)	(0.453)	(0.839)	(0.023)	(0.011)	(0.329)	(0.003)	(0.909)	(0.736)	(0.543)	(0.821)
Nursery school/Kindergarten ^b^	−0.10	−0.19	−0.10	−0.04	−0.30	−0.03	−0.18	−0.20	−0.01	−0.14
(*n* = 118)	(0.306)	(0.040)	(0.290)	(0.693)	(0.001)	(0.741)	(0.046)	(0.029)	(0.933)	(0.141)
Food intake										
Milk	−0.07	−0.13	0.11	−0.24	−0.12	−0.13	−0.08	0.04	−0.06	−0.17
(*n* = 117)	(0.484)	(0.176)	(0.259)	(0.009)	(0.187)	(0.161)	(0.380)	(0.674)	(0.499)	(0.073)
Yogurt	−0.22	−0.09	0.01	−0.23	−0.13	−0.07	−0.06	0.06	−0.20	−0.02
(*n* = 117)	(0.016)	(0.335)	(0.958)	(0.012)	(0.167)	(0.459)	(0.506)	(0.552)	(0.027)	(0.802)
Fatty fish	0.11	0.18	0.06	0.07	−0.04	0.29	0.15	0.16	0.13	−0.15
(*n* = 117)	(0.255)	(0.055)	(0.513)	(0.465)	(0.684)	(0.002)	(0.096)	(0.093)	(0.162)	(0.118)
Non-fatty fish	0.04	0.19	0.05	−0.09	−0.10	0.18	0.15	−0.00	−0.01	−0.06
(*n* = 118)	(0.677)	(0.042)	(0.632)	(0.322)	(0.294)	(0.056)	(0.115)	(0.989)	(0.956)	(0.516)
Shellfish	0.01	0.08	−0.01	−0.01	0.00	0.22	0.06	0.04	0.01	−0.07
(*n* = 118)	(0.943)	(0.387)	(0.926)	(0.904)	(0.975)	(0.020)	(0.556)	(0.680)	(0.944)	(0.469)
Green and yellow vegetables	−0.12	0.25	−0.01	0.07	−0.02	0.22	0.23	−0.07	−0.08	0.09
(*n* = 117)	(0.190)	(0.006)	(0.898)	(0.474)	(0.796)	(0.016)	(0.013)	(0.432)	(0.388)	(0.317)
Other vegetables	−0.05	0.16	−0.06	0.01	0.00	0.28	0.16	−0.14	−0.06	0.05
(*n* = 118)	(0.570)	(0.080)	(0.508)	(0.929)	(0.999)	(0.002)	(0.081)	(0.133)	(0.539)	(0.568)
Cooking water										
Tap water ^c^	0.25	−0.09	0.01	−0.16	0.16	−0.12	−0.13	0.14	0.04	−0.10
(*n* = 117)	(0.007)	(0.329)	(0.961)	(0.091)	(0.083)	(0.189)	(0.162)	(0.122)	(0.702)	(0.290)
Filtered tap water ^c^	−0.21	0.06	0.00	0.01	−0.11	0.09	0.07	−0.15	−0.06	0.08
(*n* = 117)	(0.021)	(0.522)	(0.973)	(0.925)	(0.235)	(0.325)	(0.489)	(0.112)	(0.528)	(0.371)

^a^ boy = 1, girl = 0; ^b^ Kindergarten = 1, Nursery school = 0; ^c^ yes = 1, no = 0.

**Table 5:** Mean and standard deviation in the hair of boys and girls in Japanese children.

Element	Boys vs. Girls		*p* Value (*t*-Test)	Nursery School vs. Kindergarten		*p* Value (*t*-Test)
	(*n* = 68)	(*n* = 50)		(*n* = 42)	(*n* = 76)	
Al (μg/g)	7.78 ± 5.34	12.49 ± 9.96	0.000	11.24 ± 10.23	8.97 ± 6.30	0.306
Ca (μg/g)	332.90 ± 271.80	369.87 ± 281.99	0.662	390.17 ± 301.64	325.57 ± 259.27	0.024 ^b^
Cd (μg/g) ^a^	0.02 ± 0.06	0.02 ± 0.11	0.141 ^b^	0.02 ± 0.08	0.02 ± 0.09	0.758
Cu (μg/g)	11.34 ± 7.00	14.68 ± 9.09	0.014	13.59 ± 9.67	12.29 ± 7.09	0.693
Fe (μg/g)	6.91 ± 1.94	9.31 ± 5.67	0.013 ^b^	9.39 ± 5.11	7.13 ± 3.25	0.001
Hg (μg/g)	1.06 ± 0.65	0.99 ± 0.60	0.679	1.05 ± 0.62	1.02 ± 0.64	0.741
Mg (μg/g)	34.11 ± 31.05	36.15 ± 28.93	0.737	39.72 ± 33.48	32.35 ± 27.88	0.046
Na (μg/g)	267.79 ± 610.06	106.16 ± 122.86	0.006	204.56 ± 301.50	195.44 ± 549.23	0.029
Pb (μg/g)	1.31 ± 1.11	1.80 ± 2.83	0.987	1.48 ± 1.98	1.54 ± 2.06	0.933
Zn (μg/g)	51.65 ± 17.05	59.12 ± 35.72	0.665 ^b^	59.92 ± 32.79	52.00 ± 22.46	0.141

^a^ Values below the detection limit were set at half of the detection level and treated as real values. ^b^ *p* Values from Welch’s *t*-test.

**Table 6:** Multiple regression analysis predicting the log-transformed hair Al and Pb concentrations in children’s hair.

Variables	B	SE B	*β*	*p* Value
Al (adjusted R^2^ = 0.172)				
Sex ^a^	−0.16	0.04	−0.30	0.001
Age	0.01	0.03	0.05	0.591
Yogurt consumption	−0.11	0.05	−0.21	0.015
Tap water in cooking ^b^	0.13	0.05	0.22	0.011
Pb (adjusted R^2^ = 0.050)				
Sex ^a^	0.01	0.07	0.01	0.888
Age	−0.08	0.04	−0.18	0.047
Yogurt consumption	−0.15	0.07	−0.19	0.037

^a^ boy = 1, girl = 0; ^b^ yes = 1, no = 0.

**Table 7:** Summary of studies reporting trace elements’ concentrations in hair of children from other countries (μg/g).

	Age (Years)	*n*	Al	Ca	Cd	Cu	Fe	Hg	Na	Mg	Pb	Zn
This study ^b^	3–6	118	**8.17**	279.52	0.001	**9.95**	**7.09**	0.88	*88*	25.83	0.96	51.07
Non-polluted area												
Tokyo, Japan (male) ^b^, [42]	37.9 ± 15.6	1008	6.3	**500**	0.11	**10**	13	*4.50*	*240*	**50**	2.60	**150**
Fukui, Japan ^c,d^, [43]	2–80	457	13.3	700	0.28	10.7	15.0	2.2	153	127	3.62	114
Korea ^b^, [5]	3–6	655	8.08	195.6	0.07	11.84	**11.82**	0.43	*22.44*	10.13	*1.43*	*66.23*
Germany ^a^, [20]	6–14	736	*6.57*	**269**	*0.048*	**14.9**	NA	NA	NA	**16.6**	*1.02*	141
Rome, Italy ^b^, [44]	3–15	412	8.45	**369**	0.14	10.1	12.7	NA	NA	**20.2**	5.60	149
Palermo, Sicily, Italy ^b^, [45]	11–13	137	**6.09**	NA	0.03	19.95	NA	NA	NA	NA	0.78	**179.2**
Montalto di Castro etc., Italy ^b^, [46]	9–10	92	NA	**333**	0.039	10.3	10.8	NA	NA	**27.7**	1.12	173
Madrid, Spain ^c^, [24]	0–18	648	23.8	NA	**0.0289**	**50.8**	17.3	NA	NA	NA	1.23	131.0
Alcalá de Henares, Spain ^a^, [47]	6–9	117	**6.35**	NA	0.46	**11.27**	NA	0.82	NA	NA	1.03	64.55
Turkey ^b,d^, (non-smoking households) [48]	1–6	45	2.95	NA	0.0089	6.58	6.74	0.013	NA	NA	0.625	169.4
Seltso, Russia ^b^, [31]	8.1 ± 1.1	84	17.2	**1329**	0.12	10.1	**37.6**	0.14	*304*	**206**	1.55	135
Varna, Russia ^b,d^, [25]	14.3 ± 1.3	25	NA	523.7	0.067	7.82	10.34	NA	NA	104.7	1.96	136.3
Porto Alegre, Brazil ^b,d^, [49]	12–18	126	NA	NA	0.003	3.7	NA	NA	NA	10	0.1	59
Uruguay ^c^, [50]	0.5–3	180	NA	NA	0.28	NA	NA	NA	NA	NA	17.64	NA
Bolivia (downtown) ^a^, [51]	7.6 ± 0.5	71	NA	NA	0.08 ^d^	6.89 ^d^	NA	0.15 ^d^	NA	NA	*2.32*	**85.28**
Dhaka, Bangladesh ^b^, [52]	9–10	207	NA	**521**	0.029	8.9	72	NA	NA	**64**	1.6	154
Kingdom of Saudi Arabia ^c,d,f^, [53]	6.25 ± 2.31	25	7.03	320.49	0.06	19.69	11.02	0.30	NA	70.21	0.01	149.86
Australia ^c,d,e^, (children in flood area) [54]	4	75	18.53	365.93	0.06	38.48	12.36	0.43	349.03	55.20	1.92	145.86
Polluted area												
China (mining area) ^b,d^, [55]	12.37 ± 2.21	549	NA	NA	0.10	NA	NA	NA	NA	NA	4.19	211.52
Russia (toxic waste disposal) ^b^, [31]	7.6 ± 1.3	82	19.5	**541**	**0.11**	10.4	46.2	0.09	*194*	**53.5**	**2.48**	107
Bolivia (mining area) ^a^, [51]	7.9 ± 1.0	60	NA	NA	0.07 ^d^	11.37 ^d^	NA	0.49 ^d^	NA	NA	*14.08*	**123.66**
Russia (cooper smelter) ^b,d^, [25]	14.7 ± 1.1	46	NA	217.0	0.118	11.27	15.13	NA	NA	27.9	5.44	166.0
Mt. Etna, Italy ^b^, [26]	11–13	376	4.90	NA	0.01	13.3	11.2	NA	NA	NA	0.58	199
Iglesias, Italy (mine sites) ^a^, [27]	11–13	59	4.2	NA	**0.16**	**18**	13.0	NA	NA	NA	1.5	**290**
Spain (hazardous waste incinerator) ^b^, [29]	10–13	94	NA	NA	0.02	NA	NA	*0.59*	NA	NA	*0.93*	NA

^a^ Geometric mean, ^b^ Median, ^c^ Mean, ^d^ no statistical analysis for sex difference, ^e^ Mean values for boys and girls were calculated by the author, ^f^ control group. ND = not detected, NA = not available, Bold: female > male, Italic: male > female.

## Data Availability

The datasets generated during and/or analyzed during the current study are available from the corresponding author on reasonable request.
